# A case of De Garengeot hernia with appendicitis

**DOI:** 10.1093/jscr/rjaf606

**Published:** 2025-11-04

**Authors:** Rose Taylor, Tom Burton

**Affiliations:** Department of General Surgery, Nelson Hospital, Nelson 7010, New Zealand; Department of General Surgery, Nelson Hospital, Nelson 7010, New Zealand

**Keywords:** De Garengeot, femoral, appendicitis, hernia

## Abstract

De Garengeot hernias are femoral hernias containing the appendix. They are rare and so limited literature exists regarding the presentation and best management of them. We present a case of a 60-year-old female who presented with a De Garengeot hernia with associated appendicitis followed by a summary of the current literature.

## Introduction

De Garengeot hernias are femoral hernias containing the appendix. We present a case of a 60-year-old female who presented acutely with a De Garengeot hernia with appendicitis. Imaging reported an Amyand’s hernia (an inguinal hernia containing the appendix), and so, as is often the case with these hernias, her diagnosis was established intra-operatively. She was managed with an open femoral hernia repair and appendicectomy.

## Case report

A 60-year-old European female presented to the emergency department with a painful lump in her right groin. The lump had been present for 8 months. Over this time, she had two ultrasound scans, the first reported a hydrocele of the canal of nuck and the second reported an Amyand’s hernia accompanying the hydrocele of the canal of nuck. She presented acutely as the lump had become painful over the past week. She was systemically well and had no obstructive symptoms. She had no significant medical background. To examine, she was vitally stable and had mild right lower quadrant tenderness without peritonism. She had a firm tender irreducible mass in her right groin, just inferolateral to her pubic tubercle. There were no skin changes.

**Figure 1:**
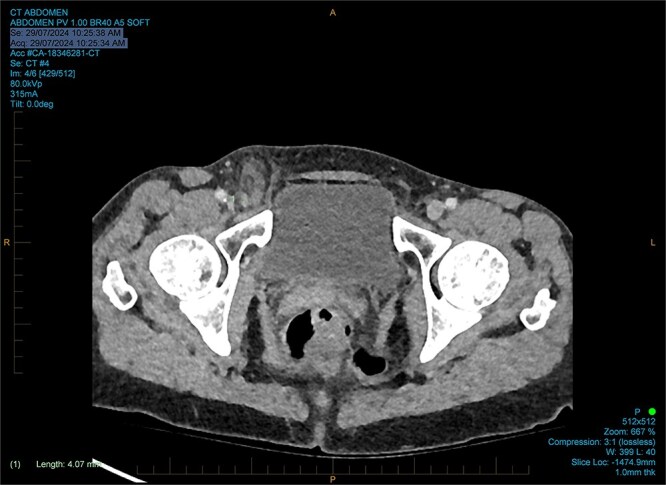
Coronal CT reported as an Amyand’s hernia.

**Figure 2:**
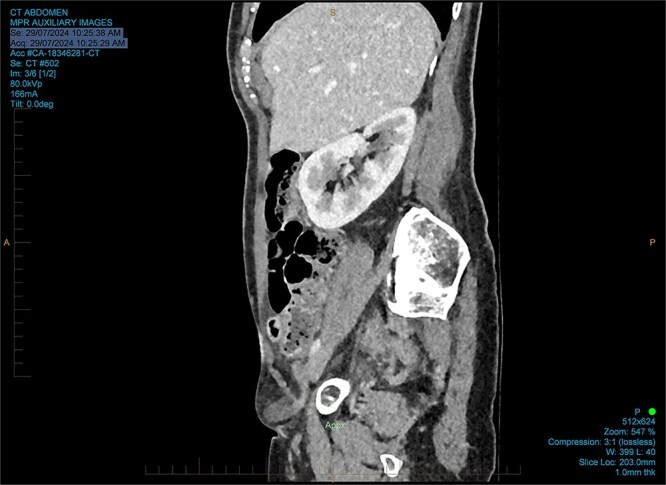
Sagittal CT reported as an Amyand’s hernia.

She had normal inflammatory markers and lactate. A computed tomography (CT) scan was reported as a direct right inguinal hernia containing the appendiceal tip (an Amyand’s hernia). There was adjacent soft tissue density and fat stranding raising the possibility of incarceration (Figs 1 and 2).

**Figure 3:**
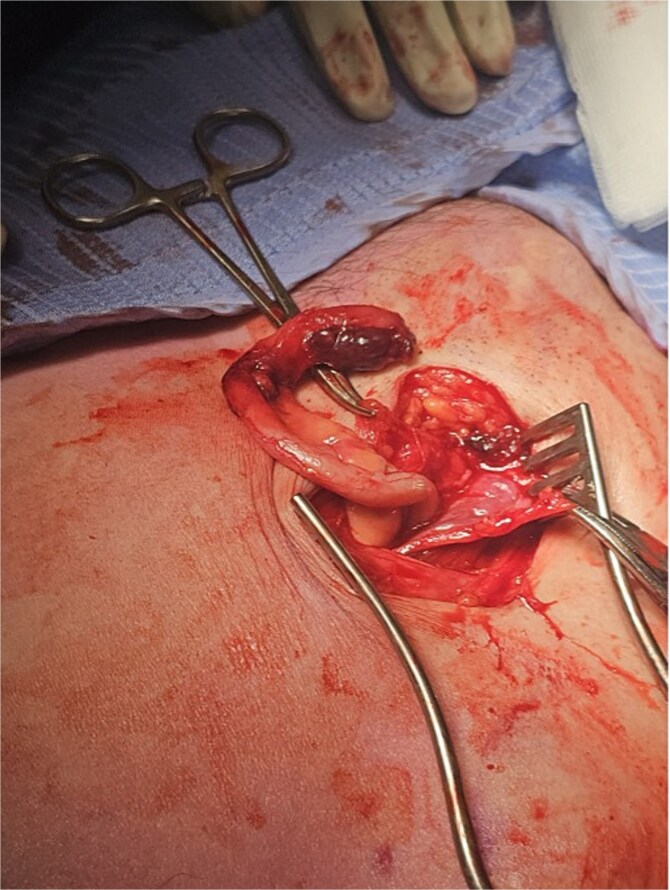
Inflamed appendix in femoral hernia.

The working radiological diagnosis was an incarcerated Amyand’s hernia. However on examination, the lump appeared to be below the inguinal ligament more in keeping with a femoral hernia. Therefore under general anaesthesia, a high approach was taken. This revealed a femoral hernia under the inguinal ligament. Rectus sheath was opened transversely. A preperitoneal reduction was unsuccessful so peritoneum was opened in the right iliac fossa. This revealed an appendix incarcerated in the femoral hernia. The hernia was reduced revealing a grossly inflamed appendiceal tip (Fig. 3). An open appendicectomy was done, the hernia was closed with an intraperitoneal 1–0 Ethibond suture before closing the peritoneum, fascia, and skin.

Histology confirmed marked ischaemia of the appendix consistent with a mechanical veno-occlusive aetiology.

She was discharged home the following day and had not experienced any complications 3 weeks post-operatively.

## Discussion

A De Garengeot hernia is a femoral hernia containing the appendix. De Garengeot hernia’s are rare, they occur in 0.5%–5% of femoral hernias and there are just over 400 cases in the literature [1, 2].

De Garengeot hernias almost always present acutely with a firm, painful, non-reducible mass inferolateral to the pubic tubercle [3]. Clinically they are indistinguishable from incarcerated femoral hernias [3]. Abdominal pain, peritonitis, and bowel obstruction are rare as the narrow femoral ring confines the inflammatory process to the hernia sac [3]. Inflammatory markers are raised in a third of cases [4].

Most, but not all, presentations of De Garengeot hernia are associated with acute appendicitis [3]. Some believe that appendicitis occurs and then migrates into the femoral hernia, while others argue that appendicitis occurs after the appendix is inside the hernia due to incarceration of the appendix [1].

Radiology can be helpful in diagnosing De Garengeot hernias but has low sensitivity. Abdominal films are non-diagnostic [3]. A meta-analysis in 2018 found ultrasound was only correct in 1/18 patients [3]. A systematic review in 2021 found CT was only 61% sensitive [2]. This is thought to be due to the rarity of De Garengeot hernias leading to misinterpretation. Despite this, CT does provide other diagnostic information (e.g. bowel obstruction or abscess). MRI is rarely used but data suggests a sensitivity of 100% [4].

Given the rarity of De Garengeot hernias, there are no clear recommendations on the best surgical management [2]. The diagnosis is most often not known prior to surgery (only in 31.5% cases) and so understandably the approach depends on the working diagnosis [2]. The general principles are repair of the hernia and appendicectomy [5]. Appendicectomy is almost always performed; however, some studies suggest that if the appendix is grossly normal it is safe to leave *in situ* [5].

The entire procedure is usually completed via a groin incision, with an open hernia repair and open appendicectomy [2]. The groin incision is most commonly an inguinal incision, although infrainguinal or suprainguinal incisions are also used [2]. Laparotomy may be required if the appendix base is unable to be accessed via the groin incision and/or for abdominal exploration [3]. The entire procedure can also be done laparoscopically, with a laparoscopic appendicectomy and laparoscopic repair of the hernia [5]. A hybrid surgery with a combined extraperitoneal and laparoscopic procedure is also possible [1]. There is no evidence that laparoscopic repair reduces hospital stay or complication rates but there are low numbers in the literature [2].

There is no clear evidence regarding repair of hernia. In an elective simple femoral hernia repair, mesh is often used. However in an acute repair of a De Garengeot hernia most cases report a primary repair with a non-absorbable suture [3]. This is due to the potential risk of contamination and subsequent mesh infection. There are some cases without severe inflammation, perforation, or abscess which have used a mesh with low infection rates [2].

In summary, De Garengeot hernias are uncommon with limited existing evidence. They typically present similar to an incarcerated femoral hernia. They can be difficult to diagnose pre-operatively due to poor radiological sensitivity. There is limited evidence about the optimal way to manage them operatively. More research is needed, in particular, in regard to the role of open versus laparoscopic repair, use of mesh, and the need for appendicectomy.
